# Avelumab mediates antibody‐dependent cellular cytotoxicity against monocyte‐derived dendritic cells through natural killer cells

**DOI:** 10.1002/mco2.70111

**Published:** 2025-02-18

**Authors:** Tatjana Sauerer, Franziska Bremm, Amke C. Beenen, Lukas Heger, Diana Dudziak, Naomi C. Bosch, Michael Erdmann, Carola Berking, Niels Schaft, Jan Dörrie

**Affiliations:** ^1^ Department of Dermatology Friedrich‐Alexander‐Universität Erlangen‐Nürnberg, Uniklinikum Erlangen Comprehensive Cancer Center Erlangen ‐ European Metropolitan Area of Nuremberg (CCC ER‐EMN) and Deutsches Zentrum Immuntherapie (DZI) Erlangen Germany

1

Dear Editor,

In recent years, treatment with immune checkpoint inhibitors (ICI) has revolutionized cancer therapy. Monoclonal antibodies that block immune checkpoint receptors such as cytotoxic T‐lymphocyte‐associated protein 4 (CTLA‐4) and programmed cell death protein 1 (PD‐1) or its ligand PD‐L1 prevent the tumor from suppressing adaptive immune responses.^1^ Especially PD‐L1 is expressed on tumor cells, but also on various healthy cell types.^2^ When we evaluated a combination therapy of active immunization via dendritic cells (DCs) and ICIs in the context of Merkel cell carcinoma (MCC) in order to improve the efficacy of the treatment, we observed an unexpected inhibitory effect of the anti‐PD‐L1 ICI Avelumab. In cell culture experiments with the most commonly used type of DCs in clinical trials on therapeutic cancer vaccination,^3^ the priming capacity of the tumor antigen‐loaded human monocyte‐derived DCs was reduced in the presence of Avelumab but not Pembrolizumab (anti‐PD‐1; Figure S1A). Experiments with GFP‐expressing DCs showed a disappearance of the DCs in co‐cultures with autologous lymphocytes and Avelumab over time (data not shown). However, no direct toxic effect of the antibody against pure DCs was observed (Figure S1B). Moreover, the DCs’ ability to stimulate T cell receptor‐transfected pure CD8^+^ T cells was not influenced by the ICI antibodies (Figure S1C).

From these unanticipated findings, we concluded that Avelumab, in the presence of autologous lymphocytes, had a detrimental effect on the DCs. In contrast to other approved anti‐PD‐L1 antibodies like Atezolizumab and Durvalumab, Avelumab contains a constant region (Fc‐part) of the IgG1 isotype that is capable of inducing antibody‐dependent cellular cytotoxicity (ADCC) against PD‐L1 expressing tumor cells, which has been shown to be beneficial in preclinical studies.^4^ However, healthy cells including DCs also express PD‐L1. Therefore, Avelumab could induce an unwanted ADCC reaction against the DCs, as any antibody with a suitable Fc‐part that binds efficiently to these cells would do.

Flow cytometry experiments showed that monocyte‐derived DCs expressed high levels of PD‐L1 and that Avelumab, Atezolizumab, and Durvalumab but not Pembrolizumab efficiently bound to the DCs (see Figure 1A). Also, the human primary DC subpopulations cDC1, DC2, and DC3, which are responsible for antigen presentation,^5^ expressed PD‐L1 upon Toll‐like receptor 7/8 stimulation with R848 (see Figure 1A). Primarily, two types of cells exert ADCC: macrophages and natural killer (NK) cells. Since the latter are present in substantial numbers in the lymphocyte fractions we used, we examined whether this was the specific cell type that carried out an Avelumab‐dependent ADCC against DCs. In a classical chromium‐release cytotoxicity assay, we were able to show that in the presence of Avelumab, DCs were efficiently lysed by purified NK cells whereas intermediate lysis was seen when complete peripheral blood mononuclear cells (PBMCs) were used as effector cells (Figure 1B). Consequently, NK‐depleted PBMCs showed no lysis. Likewise, there was also no cytotoxicity observed in the control conditions containing no antibody or the anti‐PD‐1 antibody Pembrolizumab (Figure 1B). The Fc‐part‐mutated antibodies Durvalumab and Atezolizumab were also not able to induce lysis of the DCs (Figure 1C), although they bound to the DCs to a similar extent (Figure 1A). This clearly shows that Avelumab induced an efficient ADCC reaction by NK cells against autologous DCs. In the experiments described above, monocyte‐derived cytokine‐matured DCs were used, as these cells are the most commonly used in clinical trials for DC‐based therapeutic tumor vaccination. Hence, we examined whether the maturation type and state influenced the susceptibility of DCs to Avelumab‐mediated ADCC. We observed that DCs matured with several different stimuli (cytokine cocktail, lipopolysaccharide (LPS), R848, or polyinosinic:polycytidylic acid [poly I:C]) all expressed high levels of PD‐L1. Even immature monocyte‐derived DCs expressed PD‐L1, however to a lower extent. Therefore, the cytotoxicity assay was repeated with purified NK cells and immature DCs, as well as DCs treated with the various maturation stimuli. In the presence of Avelumab all differently matured DCs and even immature DCs were lysed with similarly high efficiency (Figure 1D). This implies that the observed phenomenon may be of general relevance as it seems to apply to all cells expressing a sufficient level of PD‐L1. Next to classical ADCC, additional antibody‐mediated cytotoxic effects may exist in vivo, like complement‐dependent cytotoxicity and antibody‐dependent phagocytosis, which may aggravate the effect.

**FIGURE 1:**
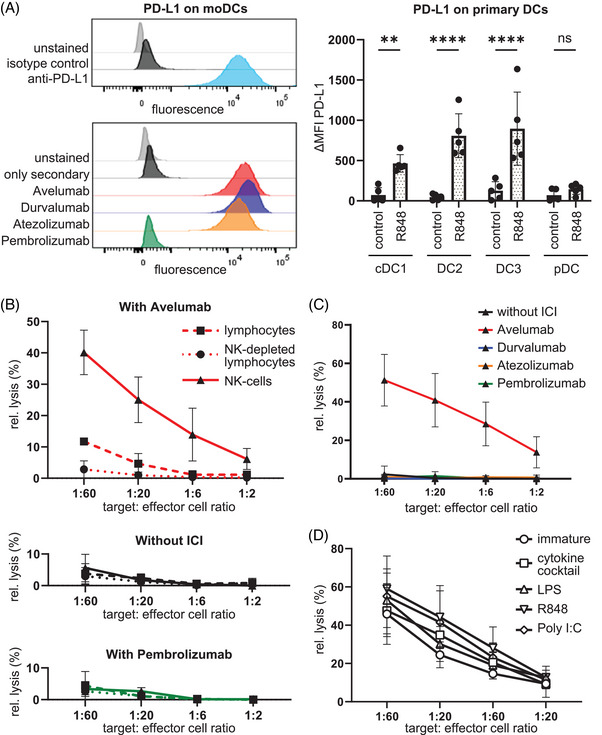
Avelumab mediates antibody‐dependent cellular cytotoxicity against programmed cell death ligand 1 (PD‐L1)‐expressing monocyte‐derived dendritic cells. (A) Monocyte‐derived dendritic cells (moDCs) were generated from the blood of healthy donors and the binding capacity of a commercially available anti‐PD‐L1 antibody, as well as the therapeutic immune checkpoint inhibitors Avelumab, Durvalumab, Atezolizumab (all anti‐PD‐L1), and Pembrolizumab (anti‐PD‐1) to the moDCs was evaluated by flow cytometry (*n* = 4; one representative donor shown). Three subtypes of conventional DCs (cDC1s, DC2s, and DC3s) and plasmacytoid DCs (pDCs) were sorted from the blood of healthy donors and stimulated with 5 µg/ml R848 for 12 h at 37°C or were control‐treated. Expression of PD‐L1 was analyzed by flow cytometry. ΔMFI values were calculated (MFI_antibody_—MFI_isotype_) and plotted as a bar graph (+SD, circles represent individual donors; *n* = 5). ** *p* < 0.01, ****p < 0.0001, ns: No significance (2way ANOVA). (B) moDCs matured with the standard cytokine cocktail (IL‐1ß, IL‐6, TNF, and PGE_2_) were used as targets in a chromium^51^‐release assay, and autologous lymphocytes (dashed lines and closed squares), NK‐depleted lymphocytes (dotted lines and closed circles) and NK cells (solid lines and closed triangles) were used as effector cells in different ratios (target:effector of 1:60, 1:20, 1:6, and 1:2). The cytotoxicity assay was performed in the presence of Avelumab (red lines), in the absence of ICI (black lines), or in the presence of Pembrolizumab (green lines). The means ± SEM of data from three independent experiments are displayed. (C): The Fc‐part‐mutated therapeutic anti‐PD‐L1 antibodies Durvalumab and Atezolizumab showed no lysis of the moDCs in the chromium^51^‐release assay. Purified NK cells were used as effector cells with target:effector ratios of 1:60, 1:20, 1:6, and 1:2. The means ± SEM of data from four independent experiments are displayed. (D): Cytotoxicity of NK cells towards either immature DCs (open circles) or DCs matured with the standard cytokine cocktail (open squares), LPS (open triangles), R848 (open inverted triangles), or poly I:C (open diamonds) in the presence of Avelumab was evaluated. Purified NK cells were used as effector cells with target:effector ratios of 1:60, 1:20, 1:6, and 1:2. The means ± SEM of data from three independent experiments are displayed. Statistics of all chromium^51^‐release assays were calculated using 2way ANOVA testing (multiple comparisons) and can be found in the supplement data S2.

In summary, we demonstrated that Avelumab‐mediated ADCC via NK‐cell activation can lead to the killing of DCs. These findings are of high clinical relevance for combination therapies with Avelumab. Therefore we recommend that patients receiving active immunotherapy like therapeutic vaccination should not simultaneously receive Avelumab treatment because this could result in an inhibitory effect on healthy PD‐L1‐expressing immune cells—especially when using *ex vivo‐*generated DCs as vaccine. Either a sequential approach with initial vaccination and subsequent Avelumab treatment, or the use of Atezolizumab, Durvalumab, or Pembrolizumab instead of Avelumab is recommended.

Avelumab is currently approved for MCC, urothelial carcinoma, and renal cell carcinoma. According to clinicaltrials.gov, Avelumab has been tested in hundreds of clinical trials from phase 1 to phase 3. Most of these combined the antibody with other treatment regimens such as chemotherapy, small molecule inhibitors, other therapeutic antibodies, oncolytic viruses, adoptive cell transfer, and therapeutic vaccination. Two trials used DCs together with Avelumab (NCT03707808 and NCT03152565) and both applied the DCs and the antibody simultaneously. Unfortunately, both did not address the question of whether the antibody had any effect on the DCs. Other trials used other types of vaccines, including peptides, adenoviral vectors, yeast formulations, and again, Avelumab was given at the same time as the vaccine. In a series of discontinued trials (QUILT‐3 series) Avelumab was given together with allogenic NK‐cells and a recombinant IL‐15 superagonist. In one trial with highly progressed MCC patients (NCT03853317), this led to serious adverse events in more than half of the patients, and in another similar trial with pancreatic cancer patients (NCT03136406), one patient reported lymph node pain as an adverse event. In a breast cancer trial (NCT04215146) with paclitaxel and an oncolytic reovirus, the additional application of Avelumab resulted in fewer clinical responses but increased serious adverse events. However, in none of these trials, the effects of the antibody on DCs were examined. Hence, we think that awareness that Avelumab can probably kill important antigen‐presenting cells will enable researchers to design more efficient treatment protocols for combination therapies with Avelumab.

## AUTHOR CONTRIBUTIONS

T.S., F.B., A.C.B., and L.H. performed experiments; T.S., F.B., and J.D. wrote the manuscript; N.C.B. and M.E. provided essential materials; T.S., J.D., N.S., and D.D. supervised experiments; A.C.B., L.H., D.D., N.B., M.E., C.B., and N.S. corrected the manuscript. All authors have read and approved the final manuscript.

## CONFLICT OF INTEREST STATEMENT

The authors declare no conflict of interest.

## FUNDING INFORMATION

This work was supported by the Deutsche Forschungsgemeinschaft (DFG, German Research Foundation) via the Research Training Group GRK2504/1 (project number 401821119), research project B2 to Diana Dudziak and B4 to Jan Dörrie.

## ETHICS STATEMENT

The blood of healthy donors was obtained following informed consent and approval of the institutional review board (Ethics Committee of the Friedrich‐Alexander‐Universität Erlangen‐Nürnberg, Erlangen, Germany: Ref. no. 4158).

## Supporting information



Supporting Information

## Data Availability

The data generated in this study are available upon request from the corresponding author.
