# Supplementary material for: Avelumab mediates antibody‐dependent cellular cytotoxicity against monocyte‐derived dendritic cells through natural killer cells
Source: MedComm (2020). 2025 Feb 18;6(3):e70111. doi: 10.1002/mco2.70111 (PMC11835948; doi:10.1002/mco2.70111)
Supplement: Supplementary file 1 — Supporting Information [file MCO2-6-e70111-s001.pdf]

Figure S1

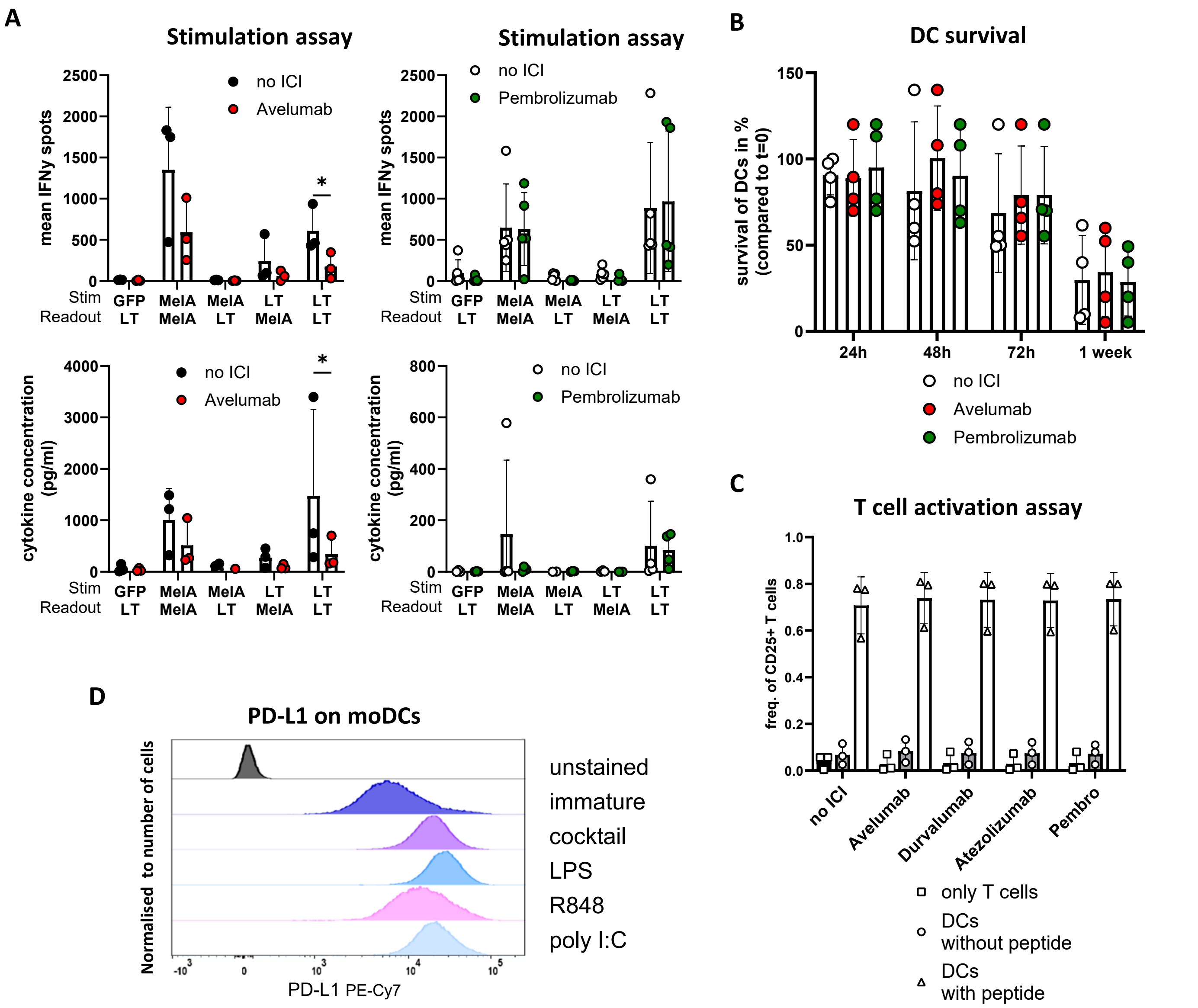

Figure S1: Effect of Avelumab on immunogenic capacity and survival of DCs. (**A**): Cytokine-matured (IL-1b, IL-6, TNF, PGE_2), monocyte-derived DCs (moDCs) were transfected with GFP, MelanA (MelA), or truncated large T antigen (LT) and co-incubated with autologous lymphocytes from healthy donors. After one and two weeks, re-stimulations were performed. The co-culture was performed without immune checkpoint inhibitors (ICI) or in the presence of Avelumab or Pembrolizumab. After three rounds of stimulation, the number of antigen-specific T cells was determined by ELISPOT with 500,000 T cells seeded in duplicates in the ELISPOT plate and the mean numbers of IFNɣ spots after the 3rd stimulation of T cells ± SEM are indicated (upper panels). The cytokine secretion of the re-stimulated T cells was determined with a cytometric bead array and the concentration of secreted IFNɣ is displayed (lower panels). The labelling of the x-axis indicates which antigen was used to load the DCs for stimulation (stim; upper line) and which was used in the read-out assay (lower line). Statistics were calculated using a 2way ANOVA (multiple comparisons) with p-values < 0.05 determined as significant (*) Number of experiments: n=3 for Avelumab, n=5 for Pembrolizumab ELISPOT, n=4 for Pembrolizumab cytometric bead array. **Avelumab significantly reduced the DC-induced specific response against LT**

(**B**): Survival of moDCs incubated without ICI, Avelumab, or Pembrolizumab over the time of 1 week. Data from DCs of four healthy donors are depicted ± SEM. The percentage of living DCs is shown compared to the number of DCs at the beginning of the experiment (t=0 h). Statistics were calculated using 2way ANOVA (multiple comparison). **Avelumab had no toxic effect on the DC**

(**C**): gp100-peptid-loaded moDCs were incubated with autologous T cells containing a corresponding gp100-TCR for 24h. The influence of the ICIs Avelumab, Durvalumab, Atezolizumab, and Pembrolizumab on the antigen presentation capability of the moDCs and the antigen-specific response of purified CD8+ T cells were analysed by flow cytometry. The MfI ± SEM of three independent experiments are shown. **None of the antibodies affected the activation T cells measured by the expression of CD25.** Similar results were obtained for CD69 (data not shown)

(**D**): moDCs were generated from blood of healthy donors and were either left immature or were matured for 24 h with the following stimuli: cytokine-cocktail (IL-1b, IL-6, TNF, and PGE_2), Lipopolysaccharide (LPS), R848, or polyinosinic:polycytidylic acid (poly I:C). The PD-L1 expression was detected by surface staining with a commercially available antibody and subsequent flow cytometry analysis. Histograms of the PD-L1 expression from one representative donor out of four are shown. **All DC preparations expressed substantial levels of PD-L1**

Appendix Methods

Cell culture, centrifugation, cell counting, cryoconservation: Cells were cultivated at 37°C, 95% humidity and 5% CO2. All steps, buffers, and media, if not otherwise indicated, were handled at room temperature (RT). T cells, peripheral blood mononuclear cells (PBMC), NK cells, and lymphocytes were centrifuged at 213 g, DCs at 140 g, for 10 min at 22°C. The Neubauer haemocytometer was used for cell counting. The procedure of cryoconservation has been described previously by Gerer et al. [1].

Generation of PBMCs, NAF, monocytes, monocyte-derived DCs, NK cells and CD8+ T cells from whole blood:
The blood of healthy donors or MCC patients was obtained following informed consent and approval of the institutional review board (ethics committee of the Friedrich-Alexander-Universität Erlangen-Nürnberg, Erlangen, Germany: Ref. no. 4158 and 4603, respectively). After isolation by density centrifugation, 3-4x10^7 PBMCs were incubated on a tissue-culture dish and after an hour the non-adherent lymphocytes (Non-adherent fraction = NAF) were separated from the adherent monocytes. The differentiation of monocytes into DCs was performed as described in detail by Lutz et al. [2] which supplements the cells cultured in DC medium (RPMI 1640, 1% heat-inactivated human AB serum, 2 mM L-glutamine, 20 mg/L gentamicin) with GM-CSF (800 U/mL) and IL-4 (275 U/mL). On day 6, the cells were left as immature DCs, or were matured either with a standard cytokine cocktail (200 IU/ml IL-1β, 1,000 U/ml IL-6, 10 ng/ml TNF, and 1 µg/ml PGE2, LPS (100ng/mL) , poly I:C (20ng/mL) , or R848 (1 µg/mL). Lymphocytes were either cryoconserved or used directly for further experiments. The NK-cell Isolation Kit was used according to the manufacturer´s instructions to separate the NK cells from PBMCs or the NAF. The isolated NK cells and the NK-depleted fraction were cultivated in MLPC medium (RPMI 1640, 10% human AB serum, 2 mM L-glutamine, 20 mg/l gentamycin, 10 mM HEPES, 1 mM sodium pyruvate, 1% MEM nonessential amino acid (100×)). A Miltenyi CD8 MACS kit was used to isolate CD8+ T cells from PBMCs according to the manufacturer's instructions. CD8+ T cells were cultivated in MLPC medium with 1 ng/ml Il-7.

RNA electroporation of cells: The transfection with RNA was performed as described by Gerer et al. [1]. Cells were washed and resuspended in Opti-MEM™ at a maximum concentration of 6 x 10^7 cells/ml of DCs or 12 x 10^7 cells/ml lymphocytes. Using a square-wave pulse, DCs were electroporated with 1250 V/cm for 1 ms and lymphocytes were transfected with 1250 V/cm for 5 ms and rapidly transferred to culture medium (DCs: DC medium with 800 U/ml GM-CSF and 250 U/mL IL-4; lymphocytes: MLPC medium with 10 ng/mL IL-7). After 2-4 hours of resting, the cells were used for further experiments. For T-cell priming and expansion, DCs were electroporated with 15 µg GFP RNA, 30 µg MelanA RNA or 30 µg truncLT RNA. All conditions were co-electroporated with 30 µg caIKKβ RNA [3]. For ELISPOT assays, stimulated lymphocytes were electroporated with 15 µg MelanA RNA or 15 µg truncLT RNA. For T cell activation assay, the CD8+ T cells were electroporated with each 15 µg RNA of the alpha and beta gp100 TCR chain [4].

Stimulation of T cells: Isolated lymphocytes were pre-stimulated with electroporated DCs for 7 days at a ratio of 20:1 (lymphocytes:DC) in MLPC medium (with IL-2 (1000 U/mL) and IL-7 (10 ng/mL) at day 2 and 4). Pembrolizumab and Avelumab were added to a final concentration of 2 µg/ml. A re-stimulation of the previously stimulated lymphocytes with freshly electroporated DCs (2nd stimulation) and with thawed DCs (3rd stimulation) followed the same protocol as the first stimulation.

IFNɣ ELISPOT assays: The quantification of the number of antigen-specific T cells was performed with an IFNɣ ELISPOT assay as described by Prommersberger et al. [5]. Pre-stimulated T cells were electroporated with MelanA or truncLT RNA and harvested after 4 h for reciprocal stimulation. The ELISPOT plate was loaded with the electroporated T cells (100.000 or 500.000 cells / well) and incubated overnight. After washing, blocking, and staining, the IFNɣ-spots were visualized and the plate was analyzed using the AID ELISPOT Reader and the AID ELISPOT Reader software V5.

Cytokine secretion by T cells: The quantification of the amount of secreted cytokines by antigen-specific T cells was determined with the BD Cytometric Bead Array human Th1/Th2 CBA Kit II. Pre-stimulated T cells were electroporated with MelanA or truncLT RNA and incubated overnight. The supernatant of the T cells was then used to analyze the cytokine secretion with the FACSCanto II flow cytometer and the FCS Express 5 Flow software.

PD-L1 expression and Avelumab binding on DCs: DCs were harvested, washed in FACS buffer (DPBS with 1% FCS and 200 mg/l sodium azide), and incubated for 30 min at 4 °C PE-Cy7 anti-human PD-L1 or the PE-Cy7 Mouse IgG1 isotype control as conjugated antibodies or with the therapeutic antibodies and the FITC-goatF(ab)2 IgG as secondary antibody for 20 min at 4 °C. Then all washed and re-suspended cells were analyzed with a FACSCanto II flow cytometer and FlowJo10 software.

T cell activation assay: moDCs were generated and matured from a healthy HLA-A02:01 positive donor and were loaded with the gp100 YLE peptide (10µg/ml) for 1h at 37°C [4]. On the previous day, CD8+ T cells were isolated from PBMCs from the same donor and electroporated with the gp100 T-cell receptor 3h prior to the incubation with the peptide-loaded moDCs. The different ICIs were added to a final concentration of 2 µg/ml. The co-culture was analysed for T-cell activation with the antibodies FITC-CD8, APC-CD69 and PE-CD25 via flow cytometry by the FACSCanto II flow cytometer and FlowJo10 software.

Cytotoxicity assay: DCs were labelled with 1 µCi Na2 51CrO4/µl of cells (20 µl 51Cr at 1 mCi/200 µl in per 100 µl 20 % HSA). Labelled DCs were co-incubated in MLPC medium with the effector cells (purified NK cells, NAF cells, or the NK-depleted fraction from NK cell isolation) that were isolated the day before at ratios of target to effector cells of 1:60, 1:20, 1:6, and 1:2. ICIs were added to a final concentration of 2 µg/ml. Target cells incubated only in MLPC medium served as minimum values, while the maximum values were calculated by the incubation of target cells with Nonidet-P40. After 4 h at 37 °C of co-incubation, the supernatants were mixed with scintillation fluid and release of chromium in the sealed counter plates was measured by the Wallac 1450 MicroBeta plus Scintillation Counter. The percentage of cytolysis was calculated as follows: [(measured release – background release)] / [(maximum release – background release)] x 100 %.

Analysis of frequencies of primary human DCs : Frequencies of primary human DCs were analyzed by flow cytometry on PBMCs isolated either from blood of healthy donors or patients treated with Avelumab. The distinction of cDC1, DC2, DC3, and pDCs followed the guidelines from Heger et al. [6] (Table S1C). For cell identification, the cells were stained for 30 min at 4 °C. Cells were acquired using a BD LSRFortessa and analyzed using FlowJo10.

Analysis of PD-L1 expression on primary human DCs: PD-L1 expression was analyzed by flow cytometry on FACS-sorted blood DCs after stimulation with or without the TLR ligand R848. For FACS-sorting, blood DCs were enriched with the EasySep Pan-DC Pre-Enrichment Kit as described before [6-8]. The DC-enriched cell suspension was stained in PBS + 2% human sera for 30 min on ice. Cells were washed, resuspended in PBS + 2% human sera + 0.1 mg/ml 4',6-Diamidino-2-phenylindole, and cell-sorted using a BD FACSAria II cell sorter into cDC1s, DC2s, DC3s, and pDCs according to Table S1D. The purity of sorted cell populations was routinely above 95%. Cell-sorted DC subpopulations were cultured in DC medium II at a concentration of 2x10^5 cells/ml and stimulated with R848 (5 µg/ml) or kept in medium at 37°C for 12 h and then stained with an anti-PD-L1 antibody or a respective isotype control for 30 min on ice. After washing, cells were acquired using a BD LSRFortessa and analyzed using FlowJo10.

Statistical analysis: For statistical analysis of the ELISPOT data, the unpaired student`s t-test was performed. All data from FACS and cytotoxicity assays were evaluated via two-way ANOVA (multiple comparisons). In general, p-values are indicated as follows: *$p < 0.05$, **$p < 0.01$, ***$p < 0.001$, ****$p < 0.0001$ and all mean values are depicted +/- SEM.

Appendix: Tables

A

-ICI	1:60	1:20	1:6	1:2
lymphocytes vs. NK cells	ns	ns	ns	ns
lymphocytes vs. NK-cell depleted lymphocytes	ns	ns	ns	ns
NK cells vs. NK-cell depleted lymphocytes	ns	ns	ns	ns

+Pembrolizumab	1:60	1:20	1:6	1:2
lymphocytes vs. NK cells	ns	ns	ns	ns
lymphocytes vs. NK-cell depleted lymphocytes	ns	ns	ns	ns
NK cells vs. NK-cell depleted lymphocytes	ns	ns	ns	ns

+Avelumab	1:60	1:20	1:6	1:2
lymphocytes vs. NK cells	*	ns	ns	ns
lymphocytes vs. NK-cell depleted lymphocytes	****	****	**	ns
NK cells vs. NK-cell depleted lymphocytes	****	****	**	ns

B

no ICI vs Avelumab	1:60	1:20	1:6	1:2
Cytokine cocktail	****	****	****	ns
PolyI:C	****	****	*	ns
R848	****	****	***	ns
LPS	****	****	**	ns
Immature	****	****	*	ns

C

Diff. ICIs vs Avelumab	1:60	1:20	1:6	1:2
No ICI	****	****	****	**
Durvalumab	****	****	****	**
Atezolizumab	****	****	****	*
Pembrolizumab	****	****	****	**

D

DC subtypes were defined as follows according to their surface marker expression	
cDC1	DAPI^{neg} HLA-DR^{pos} CD3^{neg} CD19^{neg} CD20^{neg} CD56^{neg} CD335^{neg} CD16^{neg} CD14^{neg} CD123^{neg} CD1c^{neg} CD141^{pos} CLEC9A^{pos}
DC2	DAPI^{neg} HLA-DR^{pos} CD3^{neg} CD19^{neg} CD20^{neg} CD56^{neg} CD335^{neg} CD16^{neg} CD14^{neg} CD123^{neg} CD1c^{pos} CD11c^{pos} CD5^{pos} CD163^{neg}
DC3	DAPI^{neg} HLA-DR^{pos} CD3^{neg} CD19^{neg} CD20^{neg} CD56^{neg} CD335^{neg} CD16^{neg} CD14^{neg} CD123^{neg} CD1c^{pos} CD11c^{pos} CD5^{neg} CD163^{pos}
pDC	DAPI^{neg} HLA-DR^{pos} CD3^{neg} CD19^{neg} CD20^{neg} CD56^{neg} CD335^{neg} CD16^{neg} CD14^{neg} CD123^{pos} CD1c^{pos} CD11c^{neg} CD303^{pos}

E

Characterization of surface marker expression of DC subsets for FACS-based sorting of DC-enriched cell suspension.	
cDC1	negative for CD1c, CD3, CD14, CD19, CD20, CD56, CD88, CD123, NKp46; positive for HLA-DR and CD141; intermediate for CD11
DC2	negative for CD3, CD14, CD19, CD20, CD56, CD64, CD88, CD123, CD163, NKp46, positive for HLA-DR, CD11c, CD1c
DC3	negative for CD3, CD14, CD19, CD20, CD56, CD88, CD123, NKp46; positive for HLA-DR, CD11c, CD1c, CD64, CD163
pDC	negative for CD1c, CD3, CD14, CD19, CD20, CD56, CD88, NKp46;<br>positive for HLA-DR, CD123, CD303

F

Materials	Manufacturer
lymphoprep	Axis-Shield PoC AS
DPBS-EDTA	Lonza
RPMI1640	Lonza
L-glutamine	Lonza
gentamicin	Lonza
Heat-inactivated AB serum	Sigma-Aldrich
GM-CSF	Miltenyi Biotech
IL-4	Miltenyi Biotech
IL-1β	CellGenix
IL-6	Miltenyi Biotech
TNF	Beromun, Boehringer Ingelheim Pharma
LPS	Sigma-Aldrich
Poly I:C	Sigma-Aldrich
R848	Miltenyi Biotech
HEPES	PAA Laboratories
sodium pyruvate	Lonza
MEM nonessential amino acid	Lonza
Opti-MEM™ (without phenol-red;)	Gibco
IL-2	Proleukine, Novartis
IL-7	Peprotech
Anti-IFNɣ-coated 96-well ELISPOT plates	Mabtech
DPBS	Lonza
horseradish peroxidase - conjugated anti-IFNɣ antibody	Mabtech
FCS	Sigma-Aldrich
HSA	Behring
tetramethylbenzidine substrate	
Sodium azide	Sigma-Aldrich
**Kits**	
NK-cell Isolation Kit	Milteny Biotec
EasySep Pan-DC Pre-Enrichment Kit	Stemcell Technologies
**Machines**	
Genepulser Xcell machine	BioRad
"The Big Easy" EasySep Magnet	Stemcell Technologies

Supplemental Table S2: (**A**) and (**B**) Summary of the statistical analysis of the cytotoxicity assays of lymphocytes against moDCs in presence or absence of ICIs. (**A**): For all conditions in this cytotoxicity assay, DCs were matured with the cytokine cocktail (IL-1ß, PGE2, IL-6, TNF-alpha) as used as target cells. Effector cells were either NAF, purified NK cells or NK-cell depleted NAF in different target:effector ratios (1:60, 1:20, 1:6, 1:2) in (A) the absence of ICI (-ICI) or in presence of Pembrolizumab or Avelumab. Data were obtained from blood of three healthy donors and are displayed ± SEM. Statistics were calculated using 2nd way ANOVA testing (Tukey's multiple comparisons test), ns > 0.05; *p < 0.05; **p < 0.01; ***p < 0.001; **** p < 0.0001. (**B**): For all conditions in this cytotoxicity assay, purified NK cells were co-cultured with chrom-51 labelled DCs in different target:effector ratios (1:60, 1:20, 1:6, 1:2) in the absence of ICI (-ICI) or in presence of Pembrolizumab or Avelumab. The DCs were matured with either cytokine cocktail (IL-1ß, PGE2, IL-6, TNF-alpha), PolyI:C, R848, LPS or left immature. Data were obtained from blood of three healthy donors. Here, the statistical analysis 2nd way ANOVA testing (multiple comparisons) for the conditions –ICI vs Avelumab was done for all five different maturation stimuli, ns > 0.05; *p < 0.05; **p < 0.01; ***p < 0.001; **** p < 0.0001. (**C**): For all conditions in this cytotoxicity assay, purified NK cells were co-cultured with chrom-51 labelled DCs in different target:effector ratios (1:60, 1:20, 1:6, 1:2) in the absence of ICI (-ICI) or in presence of Pembrolizumab, Avelumab, Durvalumab, or Atezolizumab. Data were obtained from blood of four healthy donors. Here, the statistical analysis 2nd way ANOVA testing (multiple comparisons) was performed for the different ICIs vs Avelumab, ns > 0.05; *p < 0.05; **p < 0.01; ***p < 0.001; **** p < 0.0001. (**D**): DC subtypes were defined as follows according to their surface marker expression. (**E**): Characterization of surface marker expression of DC subsets for FACS-based sorting of DC-enriched cell suspension. (**F**): List of materials and their manufacturers.

Appendix: References

1. Gerer, K.F., et al., *Electroporation of mRNA as Universal Technology Platform to Transfect a Variety of Primary Cells with Antigens and Functional Proteins.* Methods Mol. Biol, 2017. **1499**: p. 165-178.
2. Lutz, M.B., et al., *Guidelines for mouse and human DC generation.* Eur J Immunol, 2023. **53**(11): p. e2249816.
3. Koch, E.A.T., et al., *A One-Armed Phase I Dose Escalation Trial Design: Personalized Vaccination with IKKβ-Matured, RNA-Loaded Dendritic Cells for Metastatic Uveal Melanoma.* Frontiers in Immunology, 2022. **13**.
4. Schaft N, Willemsen RA, de Vries J, Lankiewicz B, Essers BW, Gratama JW, Figdor CG, Bolhuis RL, Debets R, Adema GJ. Peptide fine specificity of anti-glycoprotein 100 CTL is preserved following transfer of engineered TCR alpha beta genes into primary human T lymphocytes. J Immunol. 2003 Feb 15;170(4):2186-94. doi: 10.4049/jimmunol.170.4.2186. PMID: 12574392.
5. Prommersberger, S., et al., *A new method to monitor antigen-specific CD8+ T cells, avoiding additional target cells and the restriction to human leukocyte antigen haplotype.* Gene Ther, 2015. **22**(6): p. 516-520.
6. Heger, L., et al., *Guidelines for DC preparation and flow cytometric analysis of human lymphohematopoietic tissues.* Eur J Immunol, 2022: p. e2249917.
7. Hatscher, L., et al., *Circumventing pyroptosis via hyperactivation shapes superior immune responses of human type 2 dendritic cells compared to type 3 dendritic cells.* Eur J Immunol, 2023: p. e2250123.
8. Hatscher, L., et al., *Select hyperactivating NLRP3 ligands enhance the T(H)1- and T(H)17-inducing potential of human type 2 conventional dendritic cells.* Sci Signal, 2021. **14**(680).
